# Applicability of a Low-Cost, Medium-Fidelity Simulation Model to Develop Surgical Skills in Emergency Airway Management

**DOI:** 10.7759/cureus.104320

**Published:** 2026-02-26

**Authors:** Felipe Vega Rivera, Anaida Xacur Trabulce, Gessner Casas, Luis M Zamora Duarte

**Affiliations:** 1 General Surgery, Hospital Angeles Lomas, Huixquilucan, MEX

**Keywords:** advanced airway management, low-cost task trainers, simulation based training, simulation in medical education, surgical cricothyrotomy

## Abstract

Introduction: Training healthcare professionals to manage a surgical airway in emergency scenarios is essential, yet the opportunity to do so is often limited. These procedures are rarely performed, and access to high-fidelity simulation tools is generally restricted due to their cost and complexity, particularly in low-resource settings. To offer a more accessible alternative, our team developed a hybrid, medium-fidelity simulation model using porcine tracheas and synthetic skin, aiming to mimic the anatomical features of the human neck.

Methods: This prospective observational study was conducted during Advanced Trauma Life Support (ATLS) courses at Hospital Ángeles Lomas between May 2022 and January 2025. A total of 102 physicians and trainees from multiple specialties participated in structured hands-on training sessions using the simulator. The primary outcome measures were participant-rated anatomical similarity and perceived usefulness of the model for teaching both surgical and percutaneous cricothyrotomy, assessed using Likert-scale-based surveys completed immediately after the session. The simulator assembly process involved cleaning and preparing mature porcine tracheas and mounting them onto wooden bases, demonstrating feasibility and cost-effectiveness within a standardized ATLS educational setting.

Results: Of the 102 participants, the majority rated the model’s anatomy as identical or very similar to the human trachea (83/102, 81.4%). Regarding training value, 82.3% (84/102) rated the simulator as “very useful” or “ideal” for practicing both surgical and percutaneous cricothyrotomy. Importantly, no participants provided negative ratings.

Discussion: Beyond its practical function, this tool also encouraged repeated, hands-on experience in a low-risk environment. While high-fidelity models certainly offer more complex scenarios and broader learning in non-technical skills, this low-cost alternative allows for the focused development of essential manual techniques.

Conclusion: By removing financial and logistical barriers, the hybrid simulator provides a realistic and reproducible option for institutions seeking to strengthen emergency airway training without overextending their resources. It not only supports technical skill-building but also enhances clinical preparedness in high-stakes, time-sensitive situations where every second counts.

## Introduction

Emergency surgical management of upper airway obstruction is a challenge, as obstruction and loss of patency in extreme conditions carry a high cost in patients' lives. Thus, it should be an essential skill in medical training, especially for those in surgical specialties. Nowadays, various devices secure the airway to allow proper ventilation and oxygenation. However, invasive management is sometimes impossible with orotracheal or nasotracheal devices; in such cases, accessing the neck is crucial to establish a surgical airway. This is required in about 4% of cases in the emergency-trauma bay, according to an 11-year study of 9,000 patients, with an overall success rate of 90% in a 20-year systematic review [1,2].

The need to decrease risks related to the medical profession and avoid mistakes in modern surgical procedures has led specialists across several disciplines toward simulation. In 2004, Gaba mentioned simulation as a technique, rather than a technology, to replace or amplify real experiences with guided experiences. By using animate, inanimate, or cadaveric models, this technique reproduces specific aspects of the real world in an interactive and immersive way [3].

Halsted’s aphorism, proposed at the end of the 19th century, “see one, do one, and teach one,” has evolved. Currently, it is unacceptable to perform a procedure for the first time without studying, observing, and practicing it several times on simulators with anatomical qualities very similar to those of real patients. Likewise, it is unacceptable to lack the basic skills required for a procedure, even when receiving in-person mentoring.

In the current era of high-quality simulation, the concept has evolved to “see one, simulate many, demonstrate proficiency, then perform one.” This allows for learning where emotions are involved and provides a unique experience with the opportunity to practice skills by applying acquired and reflexive knowledge. Furthermore, one can cease activities at any moment and receive immediate, objective feedback.

Unfortunately, surgical practice using high-fidelity computerized models is not available at all educational levels or within the budgets of all training centers, particularly in developing countries. Empirical usage without previously developed skills leads to a constant trial-and-error model with serious consequences for patients. This situation creates a need for inanimate, low-fidelity simulators that enable surgeons and medical students to engage in affordable simulation with a reproducible reality.

Regarding invasive airway management, several attempts have been made in the simulation area, ranging from expensive manikins that are unaffordable for most institutions to animal models. Animal models may require specialized facilities with specific regulations, as well as personnel skilled in the responsible care and follow-up required by international laws for experimental animals.

The aim of this study was to present a simulator developed at the Surgical Teaching and Research Center of the Ángeles Lomas Hospital in Mexico for practicing surgical and percutaneous cricothyrotomy. 

Objectives

The primary objective was to evaluate the anatomical similarity and perceived educational usefulness of a medium-fidelity simulator for surgical airway management implemented during Advanced Trauma Life Support (ATLS) courses. The secondary objective was to assess the economic and logistical feasibility of replicating this model in clinical training programs, particularly in low- and middle-income countries.

## Materials and methods

This prospective observational study was conducted from May 2022 to January 2025 using a porcine trachea simulator model during the practical Surgical Airway Skills module of the ATLS course at Hospital Ángeles Lomas. This study was reviewed by the Investigation, Research, and Ethics Committee of Hospital Ángeles Lomas and was granted an exemption from formal ethical approval, as it involved minimal-risk educational research and anonymous post-training surveys. All participants were informed about the purpose of the study and provided implied consent by voluntarily completing the evaluation. Porcine tracheal tissue was obtained post-mortem from animals processed for commercial food purposes. No live animals were used, and no animal experimentation was performed. After completing the simulation, each medical student was invited to complete an anonymous survey evaluating the anatomical similarity of the model to the human trachea and its usefulness for developing and maintaining skills in both surgical and percutaneous cricothyrotomy.

Participants

The participants were healthcare professionals from various disciplines, including general practitioners and specialty residents in anesthesiology, general surgery, internal medicine, and critical care. Additionally, the study included attending physicians with varying levels of experience from both public and private healthcare systems in Mexico and abroad.

Inclusion Criteria

Participants included graduated medical doctors, with or without surgical specialty training, enrolled in the ATLS courses held at Hospital Ángeles Lomas between May 2022 and January 2025, who voluntarily completed the post-training survey.

Exclusion Criteria

Individuals were excluded if they had previously trained using the same simulator, were not enrolled in the ATLS course, or were not graduated medical doctors.

Simulator setup

To construct the simulator models, the following materials were required: prepared porcine tracheas, surgical instruments for dissection, a refrigerator to maintain the cold chain, wooden frames (20x25x1 cm), synthetic skin sheets (20x15x1 cm), and metal staples.

The simulator must be fully assembled prior to the training session. Additional instruments and equipment are required to access the airway, including a scalpel, dissection clamps, and a cannula.

**Figure 1:**
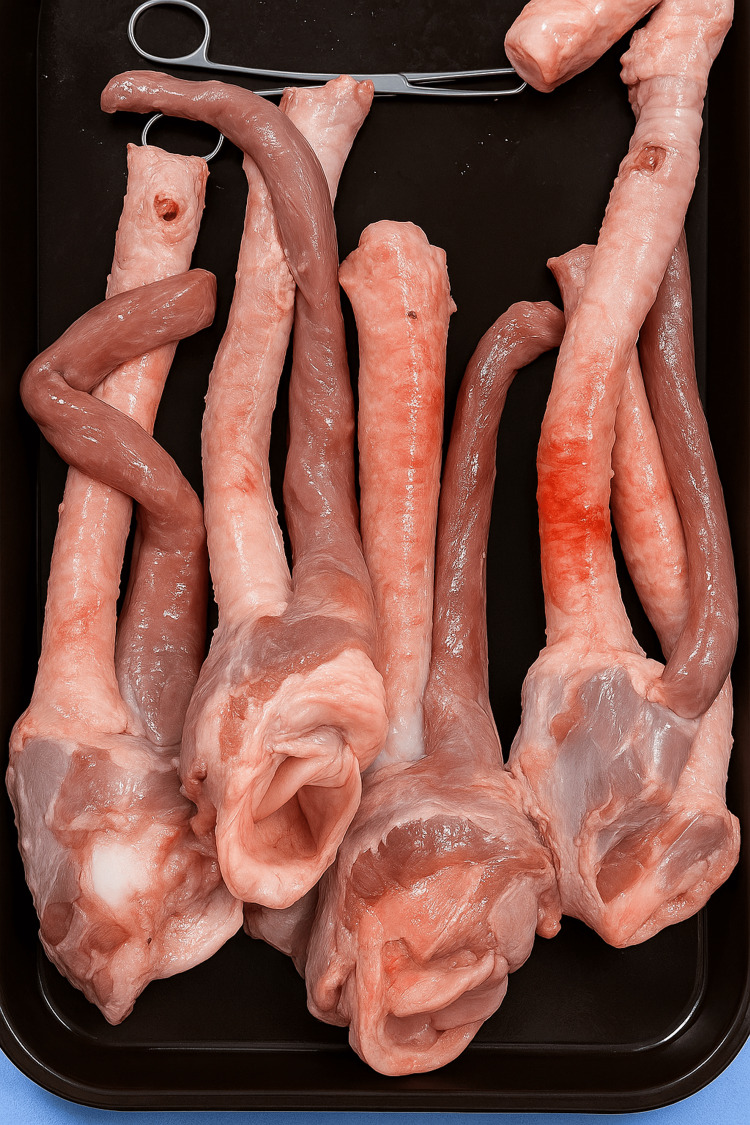
Porcine tracheas prepared for surgical airway simulation.

Each porcine trachea should include the posterior esophageal segment and intact thyroid cartilage (Figure 1).

Tracheas were prepared by dissecting the pretracheal muscles until full exposure of the cartilages was achieved. Subsequently, after a thorough wash with water, pressurized air was applied, and they were dried with environmental air for two hours. Before the final arrangement, they were cooled at 4°C for 12 hours before the training demonstration.

**Figure 2:**
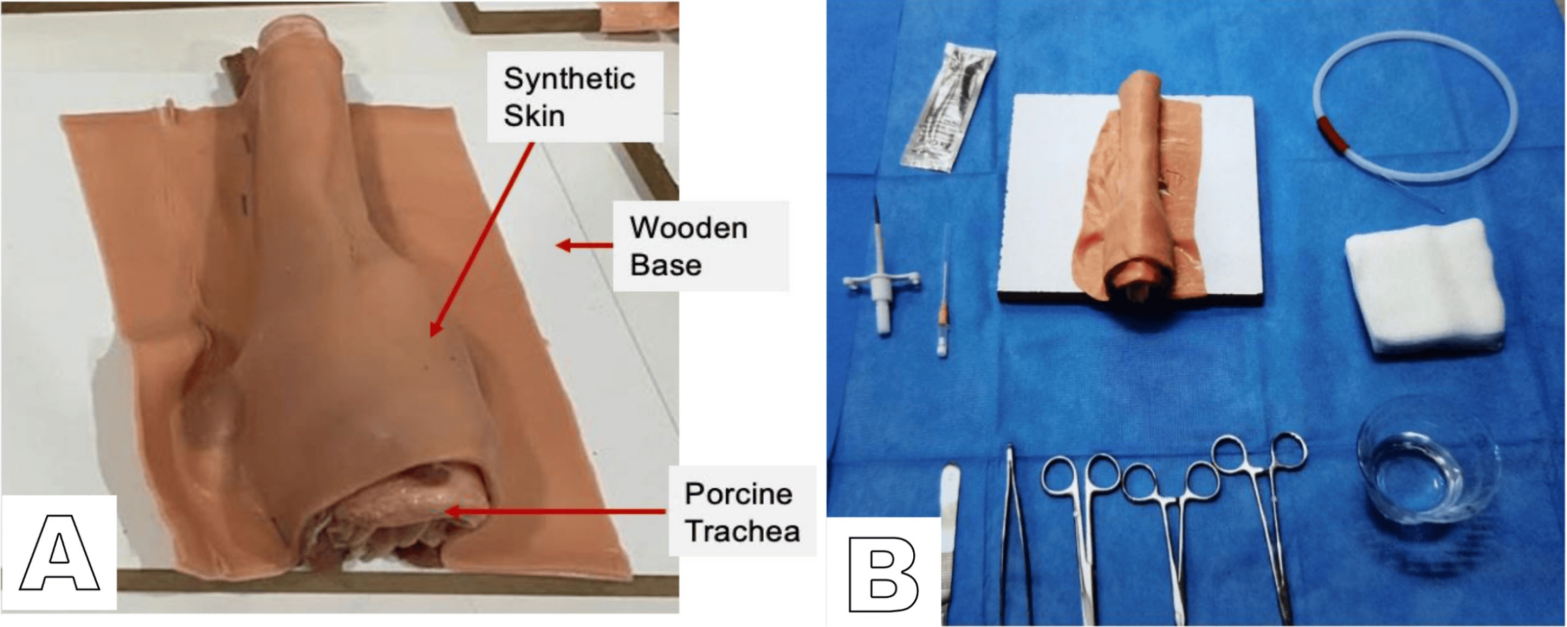
Hybrid simulation model for tracheal access training. A) Assembly of the hybrid model for tracheal simulation: The model, placed on a 25x20 cm wooden frame, is shown with a porcine trachea fixed to the surface and covered by a synthetic skin fragment. Metallic staples fix the anatomic structure, allowing for the localization of the thyroid cartilage and tracheal rings. B) Arrangement and assembly of the model: The process for ATLS demonstration is shown, including the necessary equipment to dissect soft tissues to expose the trachea completely. ATLS, Advanced Trauma Life Support

Frames of 25x20 cm and 1 cm width were prepared. Synthetic skin canvases of 20x15 cm were placed for each trachea and frame. Before the training demonstration, a clear plastic lacquer layer was applied to the anterior, posterior, and lateral sides of each trachea. Once dried, they are placed on the frame, and each trachea is covered with synthetic skin and fixed with metallic staples: one near the thyroid cartilage (main fixation), the second in the middle third of the trachea, 5 cm from the first staple, and the third in the distal portion. Finally, the same procedure is repeated on the opposite side to fix the synthetic skin thoroughly to the tracheal surface, ensuring the tracheal cartilages and cricothyroid membrane are discernible (Figure 2).

Posteriori survey 

A six-question survey was administered to participants, assessing the anatomical similarity of the medium-fidelity model to human structures and its usefulness for performing invasive airway procedures such as surgical and percutaneous cricothyrotomy (Table 1).

**Table 1:** Post-training survey questions on anatomical similarity and practical usefulness of the porcine trachea simulator model. Each item was rated on a six-point Likert scale. Anatomical similarity responses ranged from 0 (different) to five (identical); practical usefulness responses ranged from 0 (not useful) to five (ideal).

Survey domain	Survey question	Response scale
Anatomical similarity	How would you grade the appreciation of the structures in the hybrid model?	0=Different; 1=Slightly different; 2=Neutral; 3=Similar; 4=Very similar; 5=Identical
	How would you grade smoothness and structures in the hybrid model?	0=Different; 1=Slightly different; 2=Neutral; 3=Similar; 4=Very similar; 5=Identical
	How would you grade the texture of the mucosa in the hybrid model?	0=Different; 1=Slightly different; 2=Neutral; 3=Similar; 4=Very similar; 5=Identical
Practical usefulness	How would you grade the model’s usefulness for surgical cricothyrotomy?	0=Not useful; 1=A bit useful; 2=Neutral; 3=Useful; 4=Very useful; 5=Ideal
	How would you grade the model’s usefulness for percutaneous cricothyrotomy?	0=Not useful; 1=A bit useful; 2=Neutral; 3=Useful; 4=Very useful; 5=Ideal
	How would you rate the overall usefulness of the model for training in percutaneous and surgical airway techniques?	0=Not useful; 1=A bit useful; 2=Neutral; 3=Useful; 4=Very useful; 5=Ideal

Statistical analysis and data record

Descriptive statistics were used to analyze the data. Categorical variables were expressed as frequencies and percentages. Given the descriptive nature of this observational educational study, no inferential statistical tests were performed.

## Results

Preparing 16 simulators for surgical airway training takes approximately four hours and should be completed 12-24 hours prior to use.

A total of 102 participants took part in the simulation. Of these, 61.76% (63/102) stated that the anatomical structures in the hybrid model were identical to those of the human trachea, 19.60% (20/102) assessed it as very similar, and 18.62% (19/102) answered that it was similar. Importantly, none of the participants rated the model as different, slightly different, or neutral compared to human anatomical structures.

Regarding its usefulness, 53.92% (55/102) of the participants considered the model ideal, 33.33% (34/102) assessed it as very useful, and 12.74% (13/102) said it was useful for training in surgical cricothyrotomy. Meanwhile, for percutaneous cricothyrotomy, 43.13% (44/102) considered the model ideal, 35.29% (36/102) very useful, and 21.56% (22/102) useful. Regarding its general usefulness for percutaneous and surgical airway training procedures, no one considered it not useful, a bit useful, or neutral (Table 2).

**Table 2:** Anatomical similarity and usefulness of the simulation model.

Similar anatomical qualities of the model	n/102 (%)	Useful qualities of the model	Surgical cricothyrotomy, n/102 (%)	Percutaneous cricothyrotomy, n/102 (%)	General usefulness for percutaneous and surgical airway training, n/102 (%)
Identical	63/102 (61.76)	Ideal	55/102 (53.92)	44/102 (43.13)	65/102 (63.7)
Very similar	20/102 (19.60)	Very useful	34/102 (33.33)	36/102 (35.29)	19/102 (18.6)
Similar	19/102 (18.62)	Useful	13/102 (12.74)	22/102 (21.56)	18/102 (17.6)
Neutral	0	Neutral	0	0	0
Slightly different	0	A bit useful	0	0	0
Different	0	Not useful	0	0	0

Regarding the anatomical similarities of the porcine model to the human trachea, 81.36% (83/102) of the students mentioned it ranged from very similar to identical; most considered it identical, and less than a quarter evaluated it as very similar, while only 18.62% (19/102) considered it similar.

Regarding the usefulness of the model in surgical cricothyrotomy, 87.25% (89/102) said it ranged from very useful to ideal, while about one-tenth of them answered it was only useful (12.74%; 13/102). Moreover, assessing usefulness in percutaneous mode, 78.42% (80/102) thought it ranged from very useful to ideal, and almost a quarter believed it was useful (21.56%; 22/102).

When evaluating overall usefulness for both percutaneous and surgical procedures, 82.3% (84/102) of participants rated the model as very useful or ideal, while only 17.6% (18/102) considered it simply useful.

None of the interviewed students assessed the model as neutral or lower.

## Discussion

Historically, the trial-and-error model was widely accepted for acquiring surgical skills among early generations of surgeons. Nevertheless, due to the medical and ethical consequences, the concept evoked by Halsted in 1889 [4], “see one, do one, and teach one,” when he was named Surgeon-in-Chief of Johns Hopkins Hospital, has lost validity. Step by step, the way of learning new techniques has changed, and the acquisition of new knowledge through other methods, such as simulation models, has been transformed [5].

Simulation models now serve as a cornerstone of medical education, particularly for surgical specialties, as they have shown several advantages; among them, trainees can be evaluated objectively and reproducibly. This improves healthcare quality by avoiding the practice of using patients as subjects without prior proper training [6,7]. Consequently, this has improved safety and reinforced procedures, as they are repeated multiple times before involving a patient with a surgical condition in an emergency setting [8].

One of the most relevant emergency conditions is establishing a surgical airway. While infrequent, it carries high clinical risk and high responsibility for healthcare professionals; therefore, surgical residents must be qualified to manage these scenarios [9,10]. Given the rarity of these procedures, it is challenging to find training personnel with adequate experience. Thus, acquiring and maintaining the skills necessary to obtain a surgical airway in emergencies is less reliable through traditional teaching [11]. Indeed, several simulation models exist for airway skills, such as head-and-neck manikins, cadaveric models, live animal models, and a few hybrid models. Even though some are very affordable, the model proposed in this trial has a considerably lower cost and the advantage of higher anatomical similarity [12,13].

**Figure 3:**
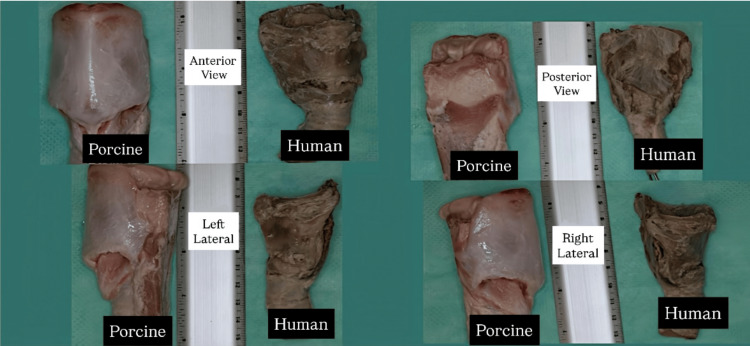
Comparison of anatomical characteristics and measurements between porcine (left) and human (right) tracheas, highlighting similarities that support their use in a simulation model. Reproduced with permission from: De la Garza H, Vega F, Hernández Y, et al. Anales de Otorrinolaringología Mexicana. 2012;57(1):17-24. This figure is reproduced with formal authorization exclusively for academic use and publication in the manuscript titled "Applicability of a Low-Cost, Medium-Fidelity Simulation Model to Develop Surgical Skill in Emergency Airway Management" for submission to Cureus. No substantial modifications were made, and full credit is given to the original authors and journal as per the permission granted by the Editor-in-Chief of Anales de Otorrinolaringología Mexicana.

Since the first publication of our laryngotracheal surgery model, we have improved the simulator by using synthetic skin instead of foam, which has provided greater realism and sensitivity. We consider it an excellent alternative for training in surgical airway management in countries with middle- and low-per-capita income [14]. In the present study, human tracheal characteristics were compared with those of the porcine trachea, and several similarities were found in the laryngotracheal portions of mature swine. Additionally, advantages of this simulator include avoiding the use of live animals, affordability, and reproducibility; it also allows for the appreciation of mucosa similar to the real tissue due to its color and hydration, as well as the anatomical similarities between porcine and human tracheas (Figure 3 and Table 3) [14].

**Table 3:** Comparison of anatomical parameters between porcine models and human specimens, including dimensions and morphological characteristics of the thyroid cartilage, cricoid, tracheal rings, vocal cords, and laryngeal ventricle. Reproduced with permission from: De la Garza H, Vega F, Hernández Y, et al. Anales de Otorrinolaringología Mexicana. 2012;57(1):17-24. This table is reproduced with formal authorization exclusively for academic use and publication in the manuscript titled "Applicability of a Low-Cost, Medium-Fidelity Simulation Model to Develop Surgical Skill in Emergency Airway Management" for submission to Cureus. No substantial modifications were made, and full credit is given to the original authors and journal as per the permission granted by the Editor-in-Chief of Anales de Otorrinolaringología Mexicana.

Structure	Parameter	Porcine model	Human specimen
Thyroid cartilage	Length (cm)	6	3.2
Thyroid cartilage	Diameter (cm)	13	9
Thyroid cartilage	Morphology	Shield-shaped; larger than human	Shield-shaped
Cricoid	Length (cm)	1.5	0.8
Cricoid	Diameter (cm)	4	1.5
Cricoid	Morphology	Not ring-shaped; similar to thyroid	Shield-shaped
Tracheal rings	Length (cm)	0.3	0.2
Tracheal rings	Diameter (cm)	6	6
Tracheal rings	Distance between rings (cm)	0.2	1.5
Tracheal rings	Morphology	Incomplete rings; similar to human	Incomplete rings
Vocal cords	Length (cm)	1.5	1.5
Vocal cords	Morphology	V-shaped; similar to human	V-shaped
Laryngeal ventricle	Diameter (cm)	0.2	0.2
Laryngeal ventricle	Morphology	Spindle-shaped; similar to human	Spindle-shaped

Moreover, this model could be adapted to train for additional procedures such as puncture cricothyrotomy, retrograde intubation, and other endoscopic airway techniques.

To enhance the training of national and international healthcare professionals and to improve simulation models, this simulator was presented at three forums prior to this publication: first, in September 2022 in Acapulco, Guerrero, Mexico, at the XXXVII Intercapitular Meeting and VIII National Meeting of Trauma (Committee for Mexico, American College of Surgeons, Chapter Distrito Federal); second, at the ATLS Global Symposium in Chicago, Illinois, USA, in March 2023 (Central Trauma Committee, American College of Surgeons); and third, at the 7th World Trauma Congress in conjunction with the 83rd Annual Meeting of the American Association for the Surgery of Trauma in Las Vegas, Nevada, in September 2024.

In terms of learning benefits, the model addresses not only cognitive objectives but also psychoaffective components, typically enhanced by high-fidelity simulators, by engaging learners emotionally and building their confidence. Furthermore, psychomotor objectives require deliberate, repeated practice, which was traditionally achieved through real patient exposure in high-risk scenarios. Medium-fidelity models were therefore developed to meet this need, providing a safer, cost-effective alternative that enables repeated and realistic training experiences.

In countries with limited budgets, or even those with comprehensive equipment for surgical education, medium-fidelity simulators are more convenient than high-fidelity ones, which require maintenance and consumables. Low-cost simulators have also been shown to facilitate the acquisition of knowledge and skills without compromising training quality. Additionally, this hybrid model, combining inanimate tissue and synthetic structures, allows beginners to understand and memorize anatomical structures, learn topographical references objectively, and build self-confidence. It also develops muscle memory and improves hand-eye coordination, as the skill can be repeated several times in a safe environment, which is crucial for more complex scenarios.

According to the literature, low- and medium-fidelity simulators are limited by a lack of interactivity and an inability to replicate the complex clinical scenarios available in high-fidelity models. Furthermore, the absence of complex situations could lead to a loss of situational learning, which is essential for training students for real-world situations. High-fidelity simulators are particularly valuable in airway emergencies, as they facilitate environmental interaction, real-time decision-making, and emotional regulation. Nevertheless, they may have lower potential regarding specific technical skill development [15]. Although the phrase "you get what you pay for" often applies, it does not necessarily hold true regarding instructional fidelity in medical simulation. Three-dimensional (3D) printing models for bronchoscopy and nasolaryngoscopy have reported positive learning results, suitability, and comfort, yet they often feature a significant cost gap; however, they remain cost-effective when considering the accuracy of anatomical details [16]. Some studies on low-cost models have demonstrated improved ability without negatively affecting training quality compared to high-cost models [17].

Regarding anatomical realism and accuracy, one limitation of low- and medium-fidelity simulators is a loss of precision that could lead to incorrect overconfidence. Some manikins exhibit anatomical inaccuracies, such as disproportionate distances between the epiglottis and the posterior pharyngeal wall, which can distort reality when using devices [18]. Realism varies between models with rough surfaces and those with more flexible, durable materials, affecting training success. Thus, these differences can cause inconsistencies in training, as replicability via touch and sight differs [19].

Nowadays, computer-aided designs for printing can detail specific aspects that may be out of proportion even in high-cost manikins, such as improper anatomical distances that vary by ethnicity or geographic location. Models developed for predominantly Caucasian populations may not reflect the anatomical proportions of individuals from Latin America, Africa, or other diverse regions. Usually, countries that produce high-fidelity models are those with higher economic and educational levels. Therefore, 3D-modeled simulators provide realism and detail, especially for structures required for skill development. However, when 3D printing first emerged, the costs of printers, materials, and software made them expensive to acquire.

In recent years, with technological advances and a competitive market, prices have decreased. Software, hardware, and platforms are now available, allowing hospitals and universities with low budgets to access this technology. Currently, it is possible to print tailor-made models with clinical scenarios and anatomical abnormalities that would otherwise be impossible to simulate. This technology represents an opportunity for psychomotor learning when high-fidelity models remain unaffordable [20]. Today, it is possible to create specific training programs for several surgical skills using lower-fidelity models, which is preferable to having no simulation-based learning at all [21].

In brief, when comparing medium-fidelity, low-cost simulators to high-fidelity versions, the former provide alternatives based on budget, availability, and effectiveness in acquiring skills and anatomical realism. The institutional budget is a primary variable, as is the type of personnel involved: medical students, clinicians, nurses, or surgeons [22]. The reduced cost of low- or medium-fidelity simulators makes them a primary option for acquiring basic skills [23,24]. A significant advantage of high-fidelity simulators is their ability to create realistic scenarios requiring complex decision-making. While the acquisition of nontechnical skills is less common in low- and medium-fidelity models, they remain relevant for learning specific technical skills [25].

Limitations

Limitations identified in this study include the following: although the human trachea shares many features with the porcine trachea, differences in size, cartilage thickness, and tissue elasticity may affect extrapolation to clinical practice. In emergency scenarios, healthcare professionals may confront physiological conditions that cannot be reproduced by the model, such as bleeding, airway edema, or patient variability. Our evaluation was based on subjective surveys, and we did not use an objective assessment of skill transfer to evaluate performance in clinical settings. Furthermore, generalizability may be limited as the study was conducted at a single institution with a small sample size. Adoption in some centers may also be restricted by the availability of biological specimens. Finally, certain aspects of training, such as decision-making under stress and team communication, can only be addressed with high-fidelity or scenario-based training. Additionally, social desirability bias may have influenced the uniformly positive participant responses, as the evaluation was based on subjective post-training surveys.

## Conclusions

The proposed medium-fidelity hybrid simulator demonstrates strong anatomical similarity, reproducibility, and affordability, making it particularly suitable for training programs in resource-limited settings. It has proven to be a valuable tool for acquiring, improving, and maintaining surgical airway management skills and is appropriate for healthcare professionals across all levels of training. Its ease of assembly and low manufacturing cost make it a practical and accessible educational tool for emergency airway procedures. By combining inanimate biological tissue with synthetic materials, this hybrid model facilitates repeated practice in a safe, realistic environment, supporting the development of essential technical competencies. While high-fidelity simulators offer enhanced realism and support for nontechnical skills, medium- and low-fidelity models remain a cost-effective and scalable alternative for core procedural training.

## References

[1] Rodríguez-Ortega MF, Delgadillo-Gutiérrez S, Basilio-Olivares A, López-Castañeda H (2003). Experiencia de 11 años en la atención del paciente politraumatizado en la Unidad de Trauma-Choque de la Cruz Roja Mexicana. An Med Asoc Med Cent.

[2] Aziz S, Foster E, Lockey DJ, Christian MD (2021). Emergency scalpel cricothyroidotomy use in a prehospital trauma service: a 20-year review. Emerg Med J.

[3] Gaba DM (2004). The future vision of simulation in health care. Qual Saf Health Care.

[4] Johnston MJ, Paige JT, Aggarwal R, Stefanidis D, Tsuda S, Khajuria A, Arora S (2016). An overview of research priorities in surgical simulation: what the literature shows has been achieved during the 21st century and what remains. Am J Surg.

[5] Cameron JL (1997). William Stewart Halsted: our surgical heritage. Ann Surg.

[6] Chapman WC (2016). Surgical training in the United States: is it time for a paradigm shift?. J Am Coll Surg.

[7] Stefanidis D, Sevdalis N, Paige J, Zevin B, Aggarwal R, Grantcharov T, Jones DB (2015). Simulation in surgery: what's needed next?. Ann Surg.

[8] Ritter KA, Horne C, Nassar A, French JC, Prabhu AS, Lipman JM (2020). Multidisciplinary simulation training improves surgical resident comfort with airway management. J Surg Res.

[9] Veenstra BR, Wojtowicz A, Walsh N, Velasco JM (2019). The emergency surgical airway: bridging the gap from quality outcome to performance improvement through a novel simulation based curriculum. Am J Surg.

[10] Altman KW, Waltonen JD, Kern RC (2005). Urgent surgical airway intervention: a 3 year county hospital experience. Laryngoscope.

[11] Andrews JD, Nocon CC, Small SM, Pinto JM, Blair EA (2012). Emergency airway management: training and experience of chief residents in otolaryngology and anesthesiology. Head Neck.

[12] Nelson MS (1990). Models for teaching emergency medicine skills. Ann Emerg Med.

[13] Pettineo CM, Vozenilek JA, Wang E, Flaherty J, Kharasch M, Aitchison P (2009). Simulated emergency department procedures with minimal monetary investment: cricothyrotomy simulator. Simul Healthc.

[14] De la Garza H, Vega F, Hernández Y (2012). Presentación de un modelo de laringe porcina para el entrenamiento en cirugía laringotraqueal asistida por endoscopia. An Orl Mex.

[15] Ernst A, Wahidi MM, Read CA (2015). Adult bronchoscopy training: current state and suggestions for the future: chest expert panel report. Chest.

[16] Pedersen TH, Gysin J, Wegmann A, Osswald M, Ott SR, Theiler L, Greif R (2017). A randomised, controlled trial evaluating a low cost, 3D-printed bronchoscopy simulator. Anaesthesia.

[17] Johnston DI, Selimi V, Chang A, Smith M (2015). A low-cost alternative for nasolaryngoscopy simulation training equipment: a randomised controlled trial. J Laryngol Otol.

[18] Blackburn MB, Wang SC, Ross BE (2021). Anatomic accuracy of airway training manikins compared with humans. Anaesthesia.

[19] Rosenthal E, Owen H (2004). An assessment of small simulators used to teach basic airway management. Anaesth Intensive Care.

[20] Yang SH, Chen CY, Liu WL, Liu HW, Chao KY (2024). Development of a cost-effective 3D-printed airway suction simulator for respiratory therapy students. Respir Care.

[21] Kennedy CC, Cannon EK, Warner DO, Cook DA (2014). Advanced airway management simulation training in medical education: a systematic review and meta-analysis. Crit Care Med.

[22] Alconero-Camarero AR, Sarabia-Cobo CM, Catalán-Piris MJ, González-Gómez S, González-López JR (2021). Nursing students' satisfaction: a comparison between medium- and high-fidelity simulation training. Int J Environ Res Public Health.

[23] Geissler ME, Bereuter JP, Geissler RB (2025). Comparison of laparoscopic performance using low-cost laparoscopy simulators versus state-of-the-art simulators: a multi-center prospective, randomized crossover trial. Surg Endosc.

[24] Srivastava A, Gibson M, Patel A (2022). Low-fidelity arthroscopic simulation training in trauma and orthopaedic surgery: a systematic review of experimental studies. Arthroscopy.

[25] Gu Y, Witter T, Livingston P, Rao P, Varshney T, Kuca T, Dylan Bould M (2017). The effect of simulator fidelity on acquiring non-technical skills: a randomized non-inferiority trial. Can J Anaesth.

